# Diquat-induced cytotoxicity on Vero and HeLa cell lines: effect of melatonin and dihydromelatonin

**DOI:** 10.2478/intox-2014-0026

**Published:** 2015-03-04

**Authors:** Roman Moravčík, Monika Okuliarová, Elena Kováčová, Michal Zeman

**Affiliations:** Department of Animal Physiology and Ethology, Faculty of Natural Sciences, Comenius University in Bratislava, Slovakia

**Keywords:** diquat, melatonin, dihydromelatonin, oxidative stress, flow cytometry

## Abstract

Diquat dibromide is a moderately toxic contact herbicide belonging to the bipyridyl group of redox-active compounds that induce a strong oxidative damage. Melatonin (MEL) can protect against oxidative damage under *in vivo* conditions, probably through its anti-oxidative capacity and ability to induce expression of anti-oxidative enzymes. The objective of this study was to investigate effects of diquat on viability of Vero and HeLa cells and possible protective effects of MEL and its analogue 2,3-dihydromelatonin (DMEL). Cell viability was evaluated with the MTT test. First, we analyzed dose-dependent effects of diquat on cell viability using the concentration range of 0.1–100 μM. Second, we used the diquat dose which reduced cell viability by 50% and treated cells with either MEL or DMEL (both in the concentration range of 1–100 μM) in the presence or absence of diquat. In addition, effects of both diquat and MEL on oxidative stress in HeLa cells were measured by flow cytometry using 2’,7’-dichlorofluorescin diacetate. We confirmed the expected negative effects of diquat on viability of Vero and HeLa cells. Melatonin and DMEL were able to prevent diquat reduced viability of Vero cells in rather low concentrations (1 μM) and DMEL exerted substantially stronger protective effects than MEL. However in HeLa cells, we did not find the same effects and MEL even reduced their viability. Moreover, treatment of HeLa cells with high concentrations of MEL (100 μM) exaggerated the pro-oxidative effects of diquat. The results suggest that in addition to the expected anti-oxidative effects, MEL exerts a pro-oxidative action which is cell type and dose dependent.

## Introduction

Minimization of negative effects of herbicides on non-target animals has been in the center of environmentally focused research for decades. To fulfill this goal, new data on physiological and pathophysiological consequences of new and approved pesticides are needed. Diquat is a redox-cycling compound used as an aquatic herbicide and belongs together with paraquat to the bipyridyl group of herbicides. Exposure of costal vegetation to diquat affects mammals, birds and their clutches (Sewalk *et al.*, 2001). Negative consequences of diquat administration result from its genotoxic and teratogenic effects (Vismara *et al.*, 2000). It is expected that the embryotoxic and teratogenic effects of both bipyridyl herbicides are manifested via induction of reactive oxygen species (ROS) by redox cycling reactions in the presence of NADPH (Bus *et al.*, 1974). However, the compounds differ in their biological effects, paraquat targeting preferentially the lung (Smith, 1987), whereas diquat causes primarily hepatic injuries (Burk *et al.* 1995). Radicals induced by the herbicides react with molecular oxygen and generate superoxide anion radicals. In addition, the superoxide radicals lead to further generation of more ROS, such as the hydroxyl radical, which then directly damages DNA (Melchiorri *et al.*, 1998).

During the last two decades, many studies have reported that the pineal hormone melatonin acts as an antioxidant and a scavenger of ROS (Reiter *et al.*, 1997; Galano *et al.*, 2011 for a review). Protective effects of melatonin were reported to exceed the protective effects of vitamin E, which belongs to the most efficient antioxidants of the intracellular compartment (Tan *et al.*, 2000). Protective effects of melatonin were reported against paraquat, a member of bispyridol herbicides (Melchiorri *et al.*, 1998), but the mechanisms of its action are still at the level of hypothesis.

The present study attempted to explore mechanisms of possible protective effects of melatonin against toxic and teratogenic effects of herbicides in *in vitro* experiments, analyzing effects of diquat on viability of the continuous and aneuploid cell line Vero, which shares characteristics of epithelial cells. Moreover, viability and oxidative stress were more precisely characterized in a human immortal HeLa cell line. In addition to melatonin, we tested the protective efficiency of the melatonin derivative 2,3-dihydromelatonin, which should be an even more effective scavenger of reactive oxygen species than melatonin (Šnirc *et al.*, 2003).

## Methods

### Cell lines and reagents

We used the Vero cell line isolated from kidney epithelium of the African green monkey (*Cercopithecus aethiops*) and the human cervical cancer cell line (HeLa) obtained from the collection of the Institute of Virology, Slovak Academy of Sciences. Cells were cultured in Dulbecco's modified Eagle's medium (DMEM; PAA Laboratories, Austria) containing glucose (4.5 g/l), penicillin (100 IU/mL) and streptomycin (100 μg/mL) supplemented with 10% (v/v) fetal bovine serum (FBS, PAA Laboratories). Cells were maintained in a humidified incubator at 37 °C with 5% CO_2_ (Heraeus, Germany). Stock solution of diquat (Zeneca Kent, UK) was prepared in phosphate buffered saline (PBS) in final concentration of 10 mM, filtered with 0.22 μm syringe filter (TPP, Switzerland) and diluted in DMEM to required concentrations. Dihydromelatonin (DMEL, obtained from the Institute of Experimental Pharmacology and Toxicology, Slovak Academy of Sciences, Bratislava, Slovakia) was dissolved in DMEM (100 mM), filtered with 0.22 μm syringe filter and subsequently diluted to required concentrations. Melatonin (Sigma Aldrich, USA) was dissolved in dimethyl sulfoxide (DMSO, Sigma Aldrich) to give a 100 mM stock solution, filtered with 0.22 μm syringe filter and further diluted in DMEM to required concentrations; the final concentration of DMSO was 1% (v/v) or less. Stock solutions of diquat, DMEL and melatonin were prepared *de novo* for each experiment.

### MTT test

The MTT assay is a colorimetric assay for assessing cell viability. Yellow MTT (3-(4,5-Dimethylthiazol-2-yl)-2,5-diphenyltetrazolium bromide, a tetrazolium salt) is reduced to purple formazan in mitochondria of living cells (Mosmann, 1983). The absorbance of this colored solution can be quantified by a spectrophotometer. Vero and HeLa cells were seeded in 96-well plate (6 × 10^3^ cells/well). After reaching 80% confluence, the medium was aspirated and Vero cells were treated by different doses of melatonin (100 μM, 10 μM, 1 μM) or DMEL (100 μM, 10 μM, 1 μM) in the presence or absence of diquat (10 μM) and HeLa cells were treated with different doses of melatonin (100 μM, 10 μM, 1 μM) in the presence or absence of diquat (100 μM). After 24 h incubation, the test compounds were aspirated and 100 μl of MTT (Sigma Aldrich) at a dilution 1:10 in DMEM (stock solution 5 mg/ml in PBS) was added. The cells were incubated in a humidified incubator at 37 °C with 5% CO_2_ for 4 h. Subsequently, MTT was aspirated and formazan was solubilized with 100 μl of 10% SDS for 10 min. at room temperature. The absorbance was measured at a wavelength of 562 nm by Microplate Reader, ELx800 with KC Junior Software (BIO-TEK Instruments Inc., USA). The percentage of live cells in different concentrations of compounds was calculated according to the formula: % of viable cells = (A_562_ sample/A_562_ control) × 100.

### Flow cytometric analysis of ROS

Intracellular ROS levels were measured by flow cytometry using 2’,7’-dichlorodihydrofluorescein diacetate (H_2_-DCF-DA, Sigma Aldrich), which easily diffuses into cells where it is cleaved by intracellular esterases to form 2’,7’-dichlorodihydrofluorescein (H_2_-DCF). This membrane-impermeable product is then oxidized to the highly fluorescent 2’,7’-dichlorofluorescein (DCF) by intracellular ROS and the redox state of the sample can be monitored by detecting the increase in fluorescence (Eruslanov & Kusmartsev, 2010). HeLa cells were plated at a density of 2.5 × 10^5^ cells per well in six-well plates. Cells were cultured in DMEM until 80% confluence was reached. In the first experiment, the cells were treated by different doses of diquat (100 μM, 10 μM, 1 μM) in the presence or absence of melatonin (100 μM) for 17 h. In the second experiment, the cells were treated by different doses of melatonin (100 μM, 10 μM, 1 μM, 0.1 μM) in the presence of diquat (10 μM) for 17 h. Each experiment consisted of 3 independent trials. After 17 h, the cells were harvested by trypsin and suspended in 1 ml phenol red-free DMEM supplemented with 2% (v/v) FBS. Then 5 × 10^5^ cells were labeled with 10 μM H_2_-DCF-DA in 0.5 ml phenol red-free DMEM for 30 min at 37°C in the dark. The cells were washed, suspended in 0.5 ml ice cold PBS and immediately analyzed for DCF fluorescence using the BD Accuri C6 flow cytometer. In each sample, 10 000 events were counted and the results were presented as fold increase of the fluorescence intensity compared with the control sample.

### Statistics

In all figures the data are expressed as mean ± standard error of mean (SEM). Statistical analyses were performed by using STATISTICA 7.0 (StatSoft Inc.). The effect of different doses of MEL and DMEL either in the presence or absence of diquat on cell viability was analyzed by two-way analysis of variance (ANOVA). The effect of different doses of diquat in the presence or absence of melatonin on ROS production of HeLa cells was analyzed with two-way repeated measures ANOVA. The effect of different doses of melatonin in the presence of diquat on ROS production was evaluated with one-way repeated measures ANOVA. Differences between individual groups were compared by Fisher's least-squares difference *post hoc* tests.

## Results

**Figure 1:**
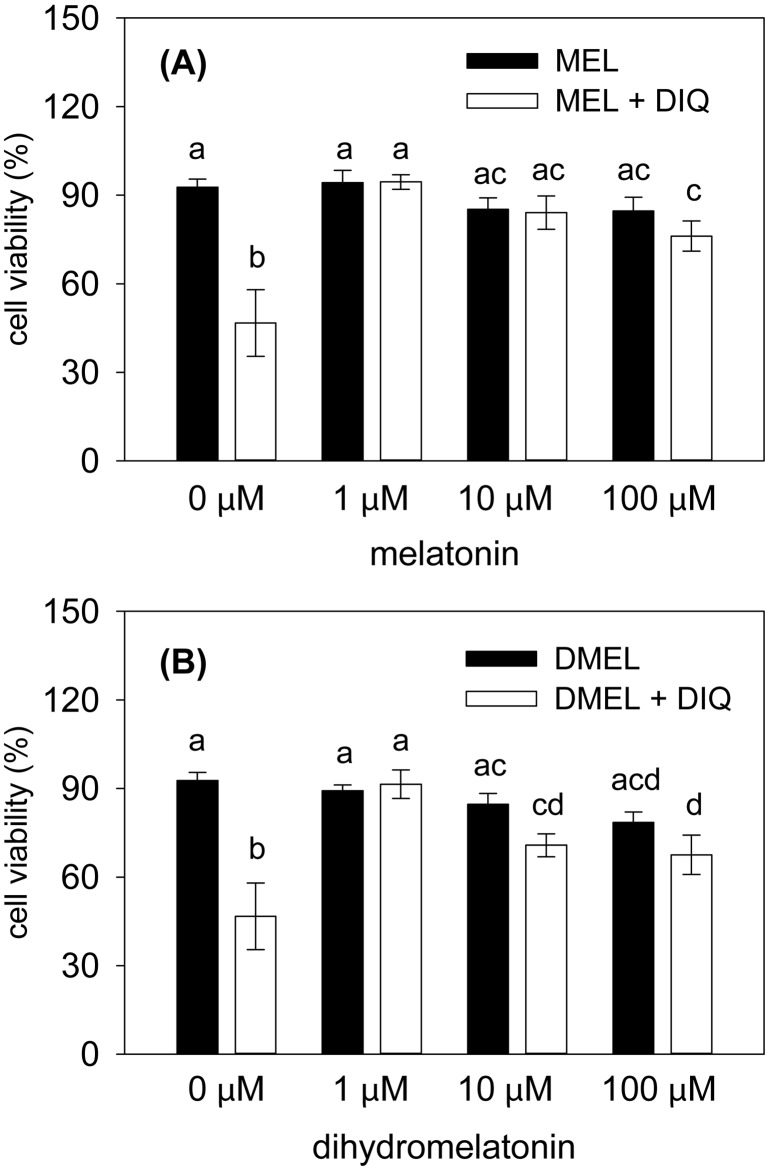
Viability of Vero cells treated with different doses of (A) melatonin (MEL) and (B) dihydromelatonin (DMEL) in the presence or absence of 10 μM diquat (DIQ) for 24 h. Cell viability was evaluated by MTT test. Data are presented as mean ± SEM (n= 5–6 per group). Different letters above columns indicate significant differences between groups at the level of *p*<0.05.

Diquat negatively influenced viability of Vero cells in a dose-dependent manner (*F*_(3,21)_=6.16, *p*<0.01; Table 1). Treatment of cells with the lowest dose of diquat (0.1 μM) did not affect cell viability, while the doses of 1 μM and 10 μM significantly reduced cell viability by 42% and 53%, respectively, as compared to the control. Thus diquat of 10 μM was used to analyze potential protective effects of MEL and DMEL on cell viability in the next experiments. Statistical analysis revealed that viability of Vero cells was significantly influenced by an interaction between diquat and MEL (*F*_(3,41)_=7.94, *p*<0.001; Figure 1A), as well as between diquat and DMEL (*F*_(3,41)_=7.17, *p*<0.001; Figure 1B). Both MEL and DMEL were effective in significantly increasing diquat-reduced cell viability in the concentration range of 1 to 100 μM. However, only the lowest and the middle dose of MEL and the lowest dose of DMEL were able to fully prevent negative effects of the herbicide on cell viability so that these groups did not differ from the control. Surprisingly, the increase of doses of MEL and DMEL did not result in an augmented protective capacity of these compounds against negative effects of diquat. On the contrary, the viability of cells treated with the highest concentration of MEL was significantly lower than that of the control. Such an inhibitory effect was seen after administration of DMEL in an even 10-times lower concentration compared with MEL.

**Table 1:** Effect of different doses of diquat on viability of Vero and HeLa cells after incubation for 24 h and assessed by the MTT test.

	Diquat (μM)				
	0	0.1	1	10	100
cell viability (%) Vero	92.7±2.8	80.3±8.7	57.9 ± 10.1**	46.7 ± 11.3***	–
cell viability (%) HeLa	100±3.4	96.9±5.2	97.8±6.4	90.2±3.2	56.4±4.0***

Values are represented as mean ± SEM (n=6 for Vero and n=15 for HeLa).

* Significant differences are expressed at the level of *p<*0.001

** *p<*0.01 as compared to the control.

**Figure 2:**
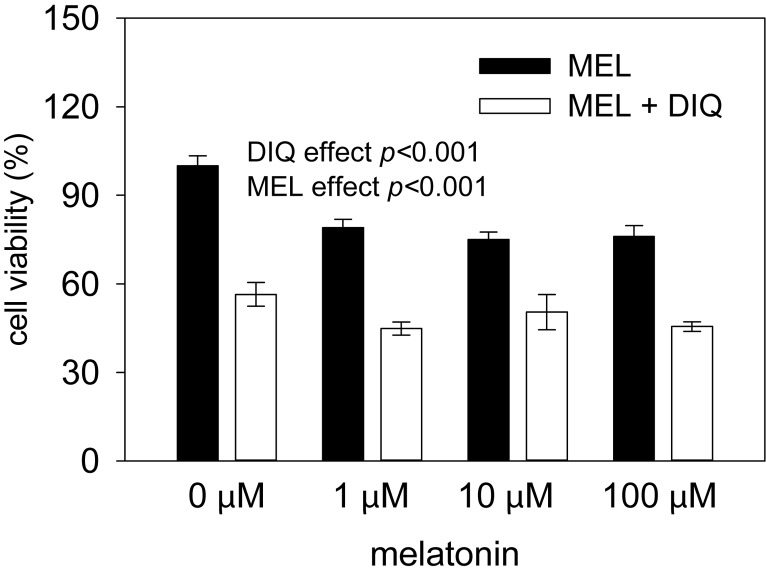
Viability of HeLa cells treated with different doses of melatonin (MEL) in the presence or absence of 100 μM diquat (DIQ) for 24 h. Cell viability was evaluated by MTT test. Data are presented as mean ± SEM (n= 3–16 per group). Statistical analysis revealed significant effect of MEL and DIQ.

Similarly to Vero cells, diquat negatively affected viability of HeLa cells in a dose-dependent manner (*F*_(4,71)_=14.98, *p*<0.001; Table 1). The highest diquat dose of 100 μM significantly reduced cell viability by 44% as compared to the control. The dose of 10 μM moderately reduced viability by 10%, while both lower doses produced no effect as compared to the control. Thus diquat of 100 μM was used to analyze potential protective effects of MEL on viability of HeLa cells in the next experiments. We found significant effects of DIQ (*F*_(1,80)_=87.34, *p*<0.001) and MEL (*F*_(3,80)_=8.59, *p*<0.001; Figure 2) without an interaction between both factors. Both compounds significantly decreased cell viability, while MEL exerted no protective effect on diquat induced cytotoxicity.

**Figure 3:**
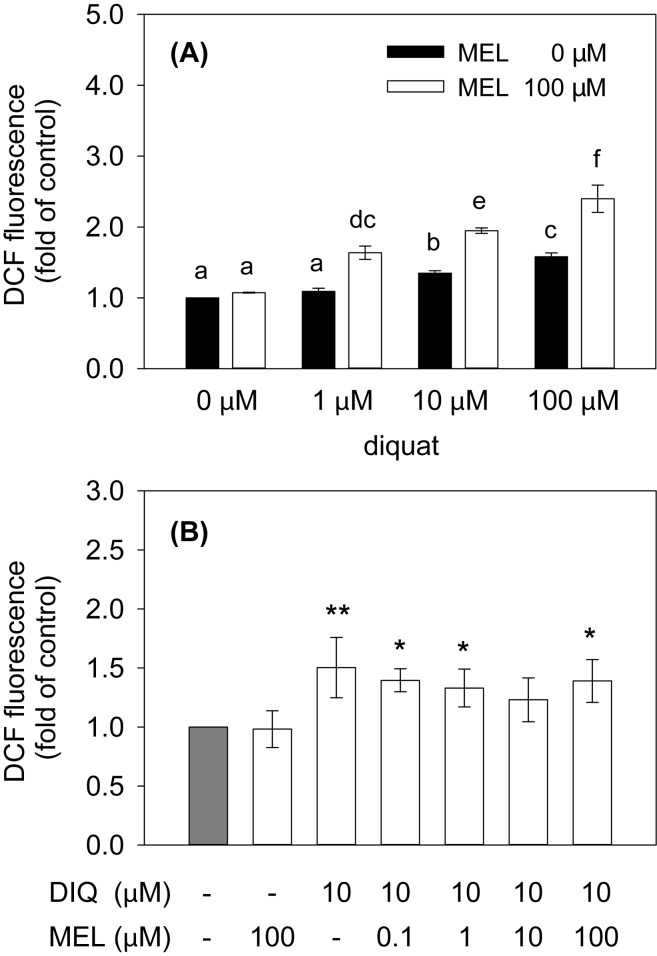
The effect of diquat (DIQ) and melatonin (MEL) on the production of reactive oxygen species (ROS) in HeLa cells. ROS production is proportional to DCF fluorescence. Bars represent mean (± SEM) of 3 trials. (A) Fold increase of DCF fluorescence in cells treated with different doses of DIQ in the presence or absence of MEL (100 μM) for 17 h. Different letters above columns indicate significant differences between groups at the level of *p*<0.05. (B) Fold increase of DCF fluorescence in cells treated with different doses of MEL in the presence of DIQ (10 μM) for 17 h. Significant differences are expressed at the level of ***p<0.001, **p<0.01 and *p<0.05 as compared to the control (grey column).

In studies of oxidative stress, ROS production was first analyzed in HeLa cells exposed to different doses of diquat in the presence or absence of melatonin (100 μM) for 17 h. We found a significant effect of an interaction between diquat and melatonin (*F*_(3,12)_=12.90, *p*<0.001; Figure 3A). Diquat increased intracellular ROS production in a dose- dependent manner, showing pro-oxidative effect of the highest (100 μM) and the medium diquat dose (10 μM). The lowest diquat dose (1 μM) did not affect ROS production as compared to the control. The high dose of melatonin (100 μM) did not influence endogenous ROS production in the absence of diquat but it enhanced the pro-oxidative effect of diquat in combination with all three diquat doses.

In the second part of the ROS production study, HeLa cells were exposed to different doses of melatonin in the presence of the medium dose of diquat (10 μM) for 17 h. We found significant differences between treatment groups (*F*_(6,12)_=4.04, *p*<0.05; Figure 3B). As compared to the control, the ROS production was elevated in cells treated with diquat while the highest (100 μM) and the two lowest (1 μM and 0.1 μM) concentrations of melatonin did not suppress this pro-oxidative effect of diquat. Treatment of cells with 10 μM melatonin and 10 μM diquat did not influence ROS production as compared to the control, indicating that melatonin in this concentration was able to suppress ROS production induced by diquat.

## Discussion

Our results confirmed the expected negative effects of diquat on viability of Vero cells in the MTT test and were in line with our previous *in vivo* studies (Zeman *et al.*, 2005). The finding that both melatonin and dihydromelatonin improved diquat deteriorated cell viability suggests that these negative effects were induced by ROS. The fact that bispyridyl herbicides induce ROS is well known (Bus *et al.*, 1974), although only paraquat was studied in detail. Moreover, recent studies suggest specific toxic effects of diquat and paraquat on the cytoskeleton of cells through interaction between microfilaments and microtubules (Vismara *et al.*, 2000). Based on these findings it can be hypothesized that protective effects of melatonin are manifested via multiple arrays of protection, including preserving microtubules and microfilaments and by their mutual interactions (King *et al.*, 2005).

Scavenging of ROS may be the prevalent mechanism and many published studies support antioxidative effects of melatonin (Galano *et al.*, 2011, for a review). These data are in line with our results obtained in Vero cells after administration of moderate doses of melatonin. More surprisingly, in HeLa cells we found pro-oxidant action of melatonin especially in high concentrations. The pro-oxidative action has been less frequently documented in scientific literature. Melatonin was found to be well tolerated and non-toxic even in very high concentrations under *in vivo* conditions. Nevertheless, some studies indicated a pro-oxidative activity of melatonin under *in vitro* conditions (Zhang & Zhang, 2014, for a review). In all cases, the pro-oxidative effects were related to very high concentrations (10–1000 μM), while lower doses (<10 μM) did not induce significant ROS generation (Wölfler *et al.*, 2001). Moreover, the pro-oxidative effects of melatonin are generally found in cancer cells and not in un-transformed cells. These cell-type-dependent effects of melatonin probably reflect differences in the control of molecular and cellular processes between normal and cancer cells but the exact mechanism is not known. Recent studies excluded the possibility of receptor mediated effects of melatonin in ROS production since the specific melatonin receptor antagonist luzindole failed to block pro-oxidative effects of melatonin (Radogna *et al.*, 2009). Surprisingly, these authors found calmodulin to be a mediator of pro-oxidative melatonin action and the mechanism has been suggested to involve metabolism of arachidonic acid. Calmodulin is a multifunctional protein and its mutual interactions with melatonin may result in activation of several intracellular pathways.

In conclusion, our results illustrate different effects of melatonin on diquat induced cytotoxicity in different cell lines, being protective in Vero cells and pro-oxidative and cytotoxic in higher doses in cancer HeLa cells. The mechanism is not yet understood and it is not clear if the pro-oxidative effects of melatonin are limited to cancer cells. The finding can be related to anti-tumor effects of melatonin yet more studies are ultimately needed in the field. Our results show that pro-oxidative effects of melatonin are cell-type and dose-dependent and can be more effective in lower than in higher doses.
